# Effects of Minor Ge on the Microstructure and Corrosion Resistance of Zn-2Al Filler Metals

**DOI:** 10.3390/ma18225111

**Published:** 2025-11-11

**Authors:** Yue Zhao, Xiaoyang Wang, Quanbin Lu, Yuanxun Shen, Yinghao Cui, Shirui Guo, Lujun Cui, Yongqian Chen, Xiaolei Li

**Affiliations:** 1 School of Mechanical & Electronic, Zhongyuan University of Technology, Zhengzhou 450007, China; 2024104013@zut.edu.cn (X.W.); laser@zut.edu.cn (S.G.); xleili@zut.edu.cn (X.L.); 2 State Key Laboratory of Advanced Brazing Filler Metals and Technology, Zhengzhou Research Institute of Mechanical Engineering, Zhengzhou 450052, China

**Keywords:** Zn-Al-Ge filler metals, intergranular corrosion, microstructure, mechanical property

## Abstract

The properties of flux-cored Zn-Al filler metals are prone to deteriorating due to corrosion, making filler metals unusable. In this study, flux-cored Zn-2Al-*x*Ge (*x* = 0, 0.3, 0.5, and 0.8 wt.%) filler metals are prepared to explore the effect of minor Ge on the corrosion resistance of Zn-2Al filler metals. The salt spray test is carried out on filler metals. A scanning transmission electron microscope is used to identify the phases in filler metals. The electrochemical performance of filler metals was tested by a workstation. The findings indicate that the microstructure of the Zn-2Al filler metal is composed of α-Al and η-Zn. Diamond-Ge forms in the microstructure of the Zn-2Al filler metal due to the introduction of Ge. Zn-2Al-*x*Ge filler metals exhibit pitting corrosion characterized by intergranular corrosion (IGC) in the salt spray environment. Ge improves the IGC resistance of filler metals by changing the distribution of α-Al in the filler metal. The Zn-2Al-0.3Ge filler metal demonstrates the most excellent corrosion resistance. It has 16% elongation after 15 d of corrosion, which is higher than that of Zn-2Al by 13.6%.

## 1. Introduction

Zinc-aluminum (Zn-Al) filler metals are employed for brazing aluminum alloys to heterogeneous materials due to their advantages, such as a low melting point, favorable wettability, and superior processability [1,2,3,4]. The flux-cored Zn-Al filler metal wire belongs to the category of composite filler metals. It is structured with a brazing flux encapsulated within a filler metal tubule. In contrast to conventional Zn-Al filler metal wires, the flux-cored Zn-Al filler metal wire demonstrates better suitability for automatic brazing equipment. Moreover, it can enhance the utilization efficiency of the flux [5,6]. However, flux-cored Zn-Al filler metals are susceptible to corrosion attacks during storage, which reduces the fluidity of filler metals [7].

Multiple approaches are employed to enhance the corrosion resistance of flux-cored filler metals [8,9,10,11,12]. Alloying element is the most common and effective method. Compared with elements such as Cu, Ti, Ag, etc., minor Ge could reduce the melting point of the Zn-Al alloy and improve its wettability [2,13]. Furthermore, minor Ge could enhance the corrosion resistance of Zinc alloys and aluminum alloys [14,15,16]. Jiang et al. [17] investigated the effects of minor Ge on the corrosion behavior of Mg-Zn alloys. The findings indicate that Ge could inhibit hydrogen evolution reactions that occur in the Zn-rich region. Mg-Zn alloys display the best corrosion resistance when the Ge content is 0.2 wt.%. Wang et al. [18] analyzed the corrosion behavior of aluminum alloys, and the findings demonstrated that minor Ge could improve the corrosion resistance of the aluminum alloy by changing the distribution of the η strengthening phase. According to patents disclosed by Cao et al. and Luo et al., Ge could decrease the corrosion current of Zn-Al alloys [19,20]. Liu et al. [14] studied the influence of Ge on the corrosion behavior of Al-Zn-Mg alloys, and the results showed that Ge decreased the densities of precipitates and improved the corrosion resistance of the Al-Zn-Mg alloy.

The effect of Ge on the corrosion resistance of the Zn-Al filler metal has not been investigated. In this study, four types of flux-cored Zn-2Al filler metals containing different Ge contents (0, 0.3, 0.5, and 0.8 wt.%) are fabricated. The accelerated corrosion process of filler metals in humid and hot environments is analyzed through the salt spray test. The effect of the trace Ge on the corrosion resistance of Zn-2Al filler metals is investigated.

## 2. Materials and Methods

In the present work, commercially pure Zinc (99.95%), aluminum (99.70%), and germanium (99.99%) are employed as raw materials to prepare Zn-2Al-*x*Ge alloys. The composition of the filler metal is listed in Table 1. Based on previous research [14,15,16,17,18], the Ge content range is 0.3–0.8 wt.%. The raw materials are loaded into a high-frequency induction graphite crucible and heated to 650 °C for 5 min. Thereafter, they are air-cooled to 25 °C to obtain the ingots. The filler metal ingot is processed into a Φ50 × 80 mm cylinder, with a Φ20 × 60 mm hole in the center. The brazing flux CsAlF4 is filled into the hole. The ingot filled with flux is extruded into alloy rods with a diameter of 8 mm at 380 °C. The alloy rods are processed into a wire-shaped flux-cored filler metal with a diameter of 3 mm through multi-pass processing.

The salt spray test is performed according to the standard GB/T10125-2021 [21]. The deionized water and NaCl are prepared in proportion to obtain a 3.5 wt.% NaCl solution. The test temperature is set to 38 °C to improve the corrosion rate. Filler metals are cleaned with ethanol before performing the salt spray test.

The electrochemical performance of Zn-2Al-*x*Ge is measured using an electrochemical workstation (CHI760E, Chenhua Instruments, Shanghai, China) in a standard three-electrode system. Prior to the test, the surface of the filler metal is sequentially ground with 400#, 800#, 1000#, and 2000# diamond abrasive papers to remove surface oxides and machining defects. All samples are washed by ultrasonic cleaning in absolute ethanol for 15 min to eliminate residual abrasive particles. After drying naturally at room temperature, samples are used as the working electrode with an exposed area of 1 cm^2^, a platinum sheet (10 mm × 10 mm × 0.5 mm) as the counter electrode, and a saturated calomel electrode (SCE) as the reference electrode. The electrolyte is a 3.5 wt.% NaCl aqueous solution (analytical grade, without further purification) and is maintained at 25 ± 1 °C throughout the test. The curves are recorded at a scan rate of 1 mV·s^−1^, with the potential range set from −1.5 V to 0.5 V (vs. SCE). Before scanning, the samples are stabilized in the electrolyte for 30 min to achieve a steady open-circuit potential (OCP). Each test is repeated three times to ensure data reliability.

The filler metal is subjected to tensile tests using a universal testing apparatus (DNS-100) (Sinotest Equipment Co., Ltd., Jinan, China) at a rate of 1 mm/min. The average tensile strength of five identical filler metals is calculated. The phases in filler metals are identified by a scanning transmission electron microscope (STEM, JEOL JEM-2100F, JEOL, Tokyo, Japan). A TEM sample with a thickness of 50 nm is prepared by focusing the ion beam (FIB, Helios G4 UX, Thermo Fisher Scientific, Waltham, MA, USA). The melting points of filler metals are determined by differential scanning calorimetry (DSC, NETZSCH DSC300, NETZSCH-Gerätebau GmbH, Selb, Germany). A scanning electron microscope (SEM, ZEISS-SUPRA55, Carl Zeiss AG, Oberkochen, Germany) and energy dispersive spectroscopy (EDS, Oxford Instruments, Oxfordshire, UK) are used to analyze the microstructure and corrosion morphology of the filler metal. The BSE mode is adopted to obtain pictures.

## 3. Results and Discussion

### 3.1. Microstructures of Filler Metals

**Figure 1:**
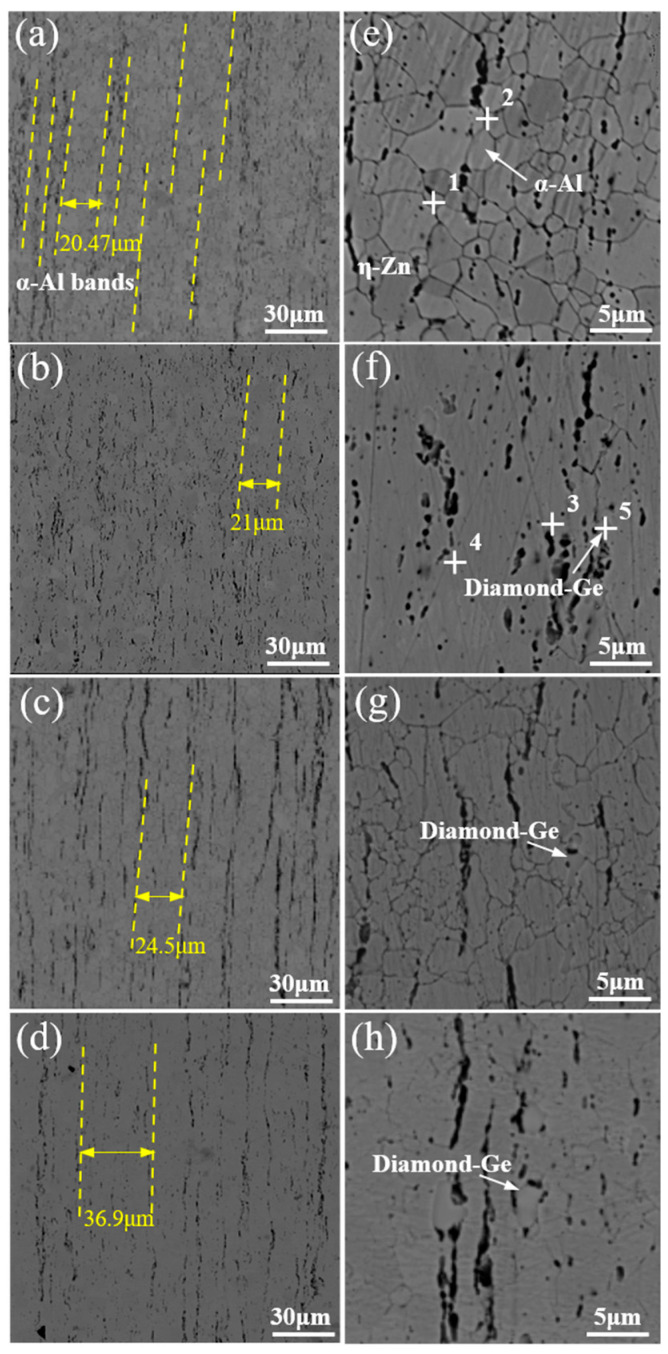
Typical microstructure of Zn-2Al-*x*Ge filler metals: (**a**,**e**) Zn-2Al; (**b**,**f**) Zn-2Al-0.3Ge; (**c**,**g**) Zn-2Al-0.5Ge; and (**d**,**h**) Zn-2Al-0.8Ge.

**Figure 2:**
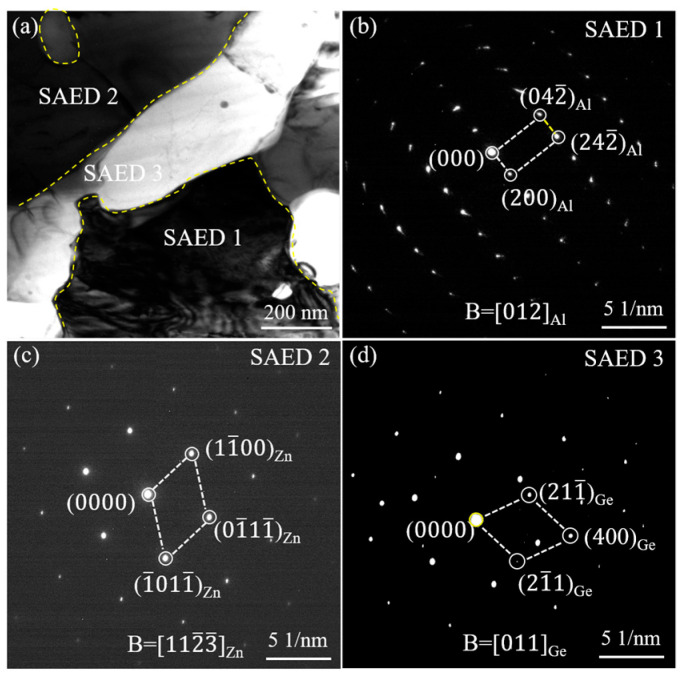
TEM analysis results for Zn-2Al-0.5Ge filler metal: (**a**) bright-field image and (**b**–**d**) selected area electron diffraction patterns corresponding to the marked locations in (**a**).

The typical microstructure of Zn-2Al filler metals with different Ge contents is illustrated in Figure 1. Zn-Al filler metals are composed of a black phase, white phase, and gray phase. According to the EDS analysis results, as shown in Table 2, the chemical composition of the black phase is 97.3 wt.% Al + 2.7 wt.% Zn. The black phase is α-Al. The chemical composition of the white phase is 3.59 wt.% Al + 96.41 wt.% Zn. The white phase is η-Zn. The chemical composition of the gray phase is 6.7 wt.% Al + 9.7 wt.% Zn + 83.6 wt.% Ge. The gray phase is represented as Diamond-Ge because it has a diamond-type atomic configuration [22,23,24]. In order to accurately characterize the crystal structure of each phase in the filler metal, a transmission analysis was carried out on the filler metal, and the results are shown in Figure 2. From the bright-field image in Figure 2a, three different areas can be found. The relevant selected electron diffraction (SAED) patterns are revealed in Figure 2b–d. SAED 1 confirmed the α-Al phase along the [012] crystal band axis of the Al crystal. SAED 2 identified the η-Zn phase along the [11-2-3] crystal band axis of the η-Zn crystal. The remaining Ge in the filler metal formed the Diamond-Ge phase in the SAED 3 region along the [011] crystal band axis of the Diamond-Ge crystal. Therefore, the black phase is α-Al, the white phase is η-Zn, and the gray phase is Diamond-Ge.

**Figure 3:**
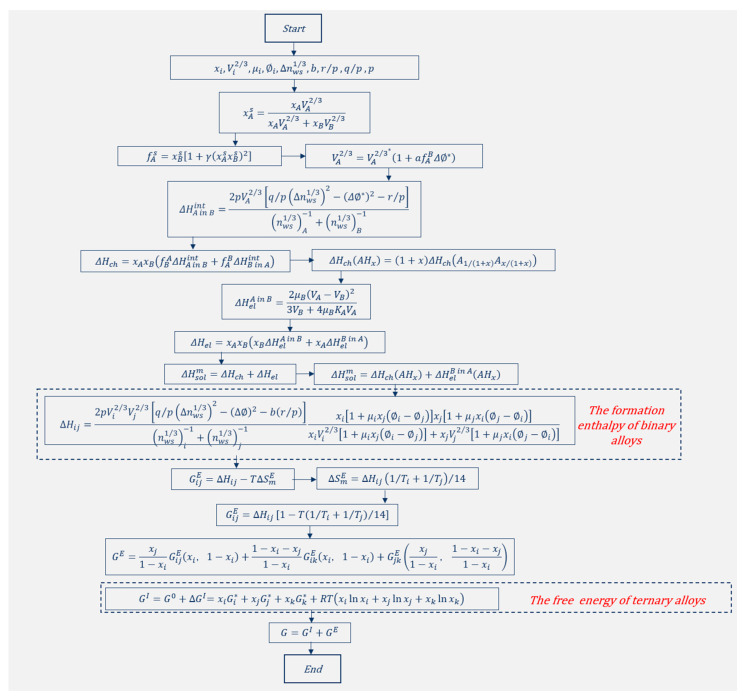
Schematic diagram showing calculation procedure for extension of Miedema model from binary to ternary system.

**Figure 4:**
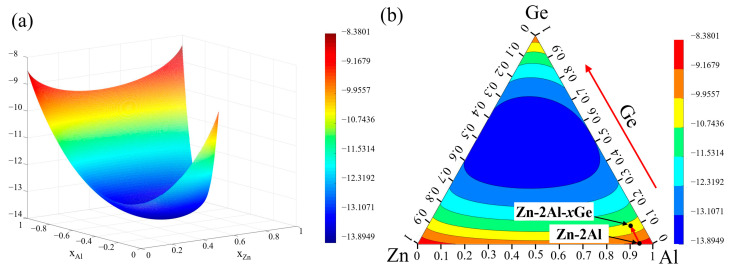
The free energy contour maps of the Zn-Al-Ge ternary alloy system at 25 °C. (**a**) three-dimensional graphs; (**b**) contour map corresponding to (**a**).

Figure 1a–d displays the distribution of α-Al in the microstructures of Zn-2Al-*x*Ge filler metals. It can be seen that the α-Al phase displays obvious band-like arrangement characteristics. Compared with the Zn-2Al filler metal, the distance of the two adjacent α-Al bands was increased from 20.47 μm to 36.9 μm. In addition, the majority of α-Al coexists with the Diamond-Ge, showing a strong spatial association. In addition, a minor amount of α-Al is sparsely dispersed within the η-Zn, with no obvious aggregation characteristic in the filler metal. As the Ge content increases, the trend of the aggregation of α-Al becomes more pronounced, and the size of the α-Al phase gradually increases. When the Ge content is 0.3 wt.%, α-Al phases aggregate and exhibit discontinuous distributions. When the Ge content is 0.8 wt.%, the α-Al gradually becomes continuously distributed. This is mainly attributed to Ge exerting a thermodynamically driven enrichment effect on Al, as this interaction aligns with the minimum free energy principle of the alloy system. The free energy of the Zn-Al-Ge ternary system is calculated based on the Miedema model of solution enthalpy, the Tamaka model, and the Toop model [24,25,26], as shown schematically in Figure 3. Figure 4 shows the free energy contour map of the Zn-Al-Ge ternary system at 25 °C. It can be seen that the ΔG is smaller when the Ge content is not zero, indicating that Ge makes it more convenient to generate ternary eutectic phases rather than binary eutectic phases in the Zn-Al-Ge ternary alloy system. This can be confirmed by the EDS analysis results in this article. Therefore, it can be inferred that in order to reduce the free energy of the system, Al will accumulate in the high-Ge region, which causes the precipitated Al to aggregate in the Diamond-Ge during the drawing process, increasing the size of the α-Al phase and promoting the continuous distribution of α-Al.

**Figure 5:**
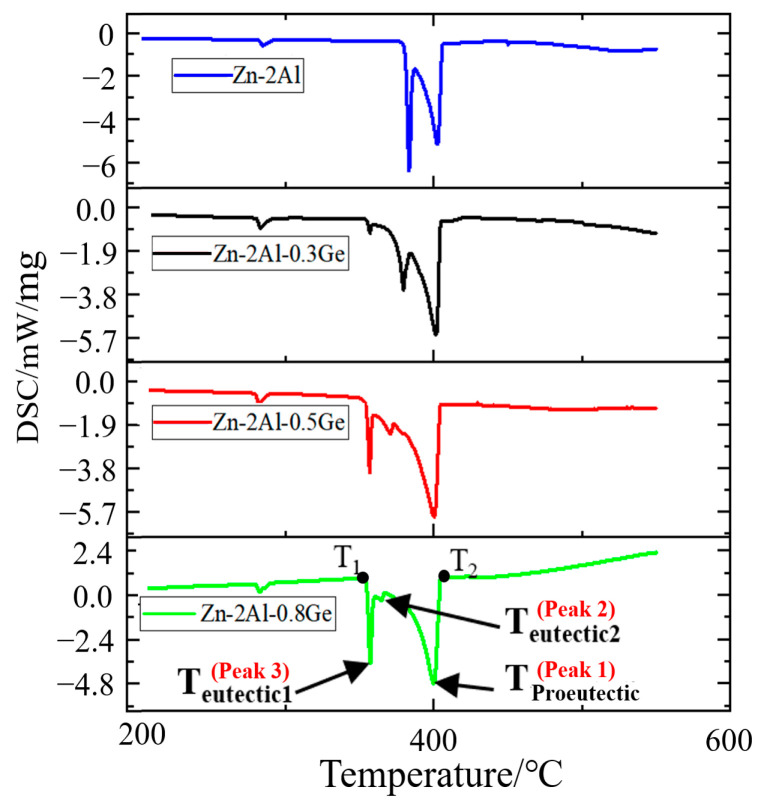
DSC curves of Zn-2Al-*x*Ge filler metals.

DSC curves of Zn-2Al-*x*Ge filler metals are displayed in Figure 5, and the relevant temperature data is shown in Table 3. It can be seen that there are mainly two peaks when the Ge content is zero. Peak 1 appears between 385 °C and 410 °C, corresponding to the precipitation of the primary Zn, and peak 2 (between 360 °C and 400 °C) represents the binary Zn-Al eutectic process. While peak 3 appears (between 345 °C and 355 °C) when the Ge content is not zero. According to the Zn-Al-Ge ternary phase diagram [27], peak 3 represents the ternary Zn-Al-Ge eutectic process. As the Ge content increases from 0 to 0.8 wt.%, peak 3 becomes sharp, and peak 2 gradually flattens, indicating the transformation from the Zn-Al eutectic reaction to the Zn-Al-Ge eutectic process. Therefore, the solidus temperature (T_1_) decreased by 28 °C. In addition, the temperature of peak 1 (T_proeutectic2_) moves left, and the liquidus temperature (T_2_) decreases from 405.61 °C to 404.27 °C, indicating that Ge can promote the precipitation of primary Zn and reduce the liquidus temperature of the Zn-2Al filler metal.

### 3.2. Corrosion Behavior Analysis

**Figure 6:**
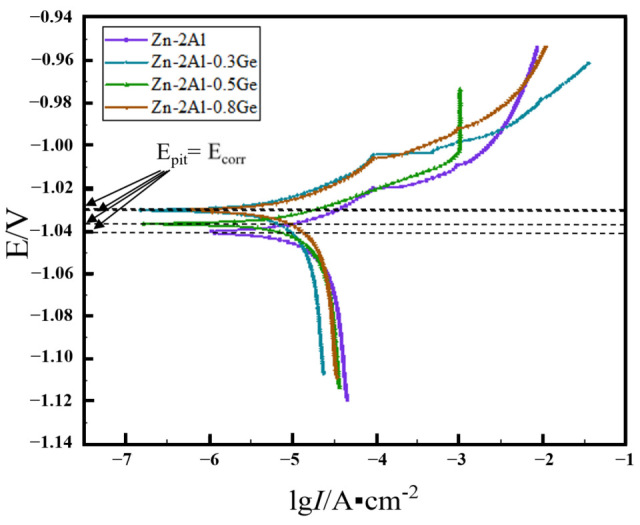
Polarization curves of Zn-2Al-*x*Ge filler metals.

Figure 6 presents the polarization curves of Zn-Al filler metals with varying Ge contents when immersed in a 3.5 wt.% NaCl solution. All filler metals exhibit polarization curves with similar features. For the cathodic polarization curves, a clear trend emerges: as the cathodic overpotential goes up, the cathodic corrosion current first rises rapidly and then increases at a slower rate. This trend suggests that the diffusion process of oxygen (O) exerts an influence on the cathodic reaction. The anodic polarization curves show a different characteristic: as the anodic overpotential increases, the anodic corrosion current rises sharply. At this stage, the pitting potential (E_pit_) equals the corrosion potential (E_corr_)—a phenomenon that indicates that the anodic reaction is affected by the pitting process.

Additionally, it can be seen from Figure 6 that the pitting potential of the Zn-2Al filler metal is −1.21 V, and it goes up when the Ge content is not zero. This change in the pitting potential means that Ge decreases the corrosion tendency of the filler metal. The polarization curves are fitted, and the resulting data shows that the corrosion currents of the four target filler metals are 1.36 × 10^−5^ A/cm^2^, 7.21 × 10^−6^ A/cm^2^, 1.06 × 10^−5^ A/cm^2^, and 1.12 × 10^−5^ A/cm^2^, respectively. This set of data indicates that the pitting current of the filler metals decreases firstly and then increases with the growth of the Ge content.

**Figure 7:**
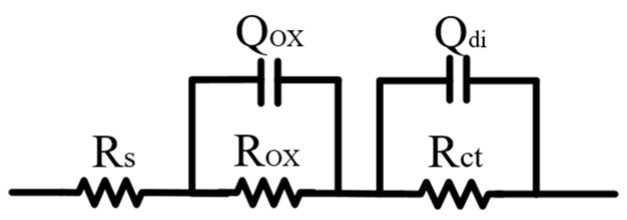
Equivalent circuit diagram of Zn-2Al-*x*Ge filler metals.

**Figure 8:**
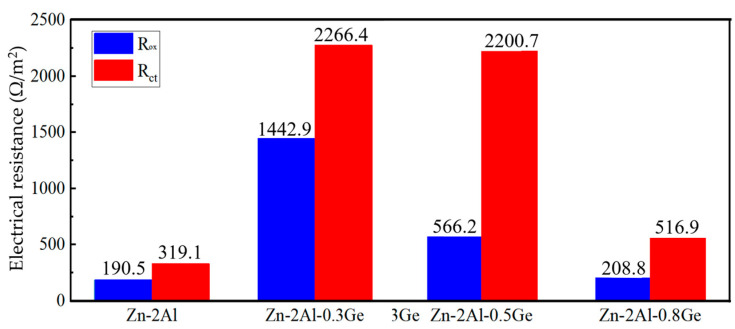
EIS fitting results of Zn-2Al-*x*Ge filler metals.

The electrochemical impedance spectroscopy (EIS) of Zn-2Al filler metals is investigated, and the obtained curves are fitted through an equivalent circuit diagram, as shown in Figure 7, in which R_s_ represents the electrical resistance of the 3.5 wt.% NaCl solution, R_OX_ represents the electrical resistance related to the oxide film, R_ct_ represents the electrical resistance related to the charge transfer, Q_OX_ represents the capacitance related to the oxide film, and Q_di_ represents the electrical double-layer capacitor. Figure 8 shows the fitting results. It can be seen that the R_OX_ and R_ct_ of the Zn-2Al filler metal is 190.5 Ω/m^2^ and 319.1 Ω/m^2^, respectively, which is lower than when the Ge content is not zero. In addition, as the Ge content increases, the R_OX_ and R_ct_ increase at first and then decrease, and the peak appears at the Zn-2Al-0.3Ge filler metal. This reveals that Ge strengthens the protective effect of the oxide film on filler metals and increases the hindrance of the charge transfer, thereby improving the corrosion resistance of filler metals.

**Figure 9:**
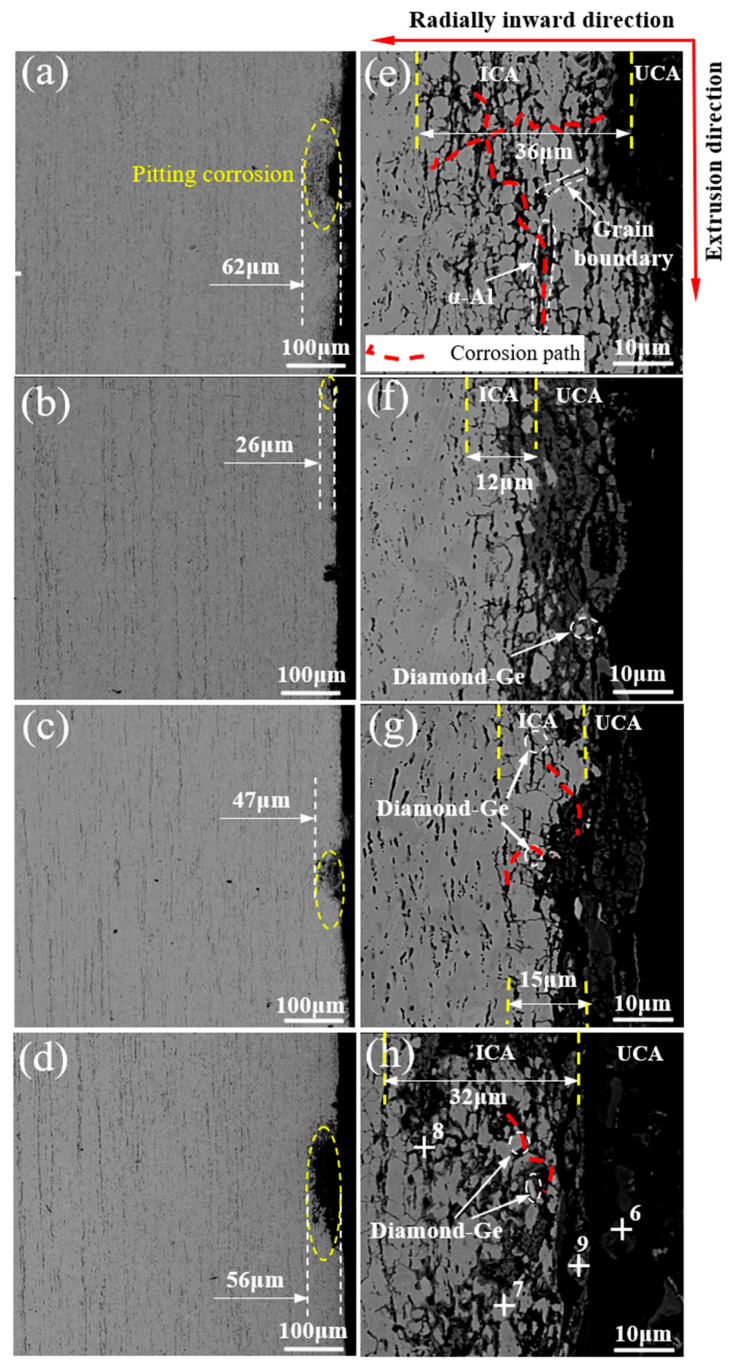
Pitting corrosion of Zn-2Al-*x*Ge filler metals: (**a**,**e**) Zn-2Al; (**b**,**f**) Zn-2Al-0.3Ge; (**c**,**g**) Zn-2Al-0.5Ge; and (**d**,**h**) Zn-2Al-0.8Ge.

Figure 9 presents the microstructure of the Zn-2Al filler metal after 10 h of salt spray corrosion. It can be seen that pitting corrosion occurs on the surface of the filler metal, and the corroded areas exhibit a distinct grid-like morphology, which is a typical characteristic of IGC. Figure 9b–d illustrate the microstructures of Zn-2Al-*x*Ge filler metals. It can be seen that the depth of corroded regions reduces when the Ge content is not zero. This demonstrates that Ge exerts a positive effect on enhancing the corrosion resistance of the Zn-2Al filler metal. However, as the Ge content increases from 0.3 wt.% to 0.8 wt.%, the corrosion depth increases accordingly.

**Figure 10:**
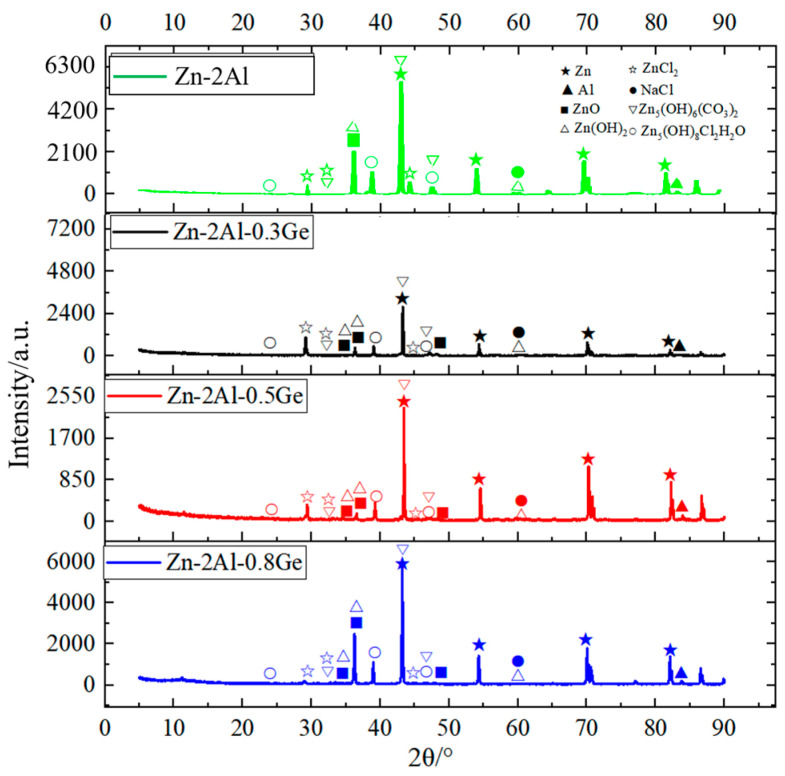
XRD patterns of Zn-2Al-*x*Ge filler metals after salt spray corrosion test.

Figure 10 illustrates the XRD pattern of corrosion products derived from Zn-2Al filler metals with varying Ge contents. The corrosion products are mainly composed of Zn(OH)_2_, ZnCl_2_, ZnO, Zn_5_(OH)_8_Cl_2_·H_2_O, and Zn_5_(OH)_6_(CO_3_)_2_. According to Table 4, for the Ge-rich region and Al-rich region, the Zn content decreased obviously, indicating that η-Zn occurs an anodic dissolution reaction. Therefore, corrosion occurring in filler metals belongs to electrochemical corrosion, and η-Zn acts as the anode.

In order to further investigate how Ge influences the corrosion performance of Zn-2Al filler metals, corrosion propagation paths are analyzed as shown in Figure 9e–h. According to Figure 9e, corrosion mainly propagates along the extrusion direction (α-Al bands) and the radially inward direction (grain boundaries of η-Zn). Compared with the radially inward direction, corrosion is more likely to extend along the extrusion direction. In addition, η-Zn grains adjacent to α-Al grains in the Zn-Al-Ge filler metal suffer more severe corrosion attacks, whereas the corrosion at grain boundaries between α-Al bands is less pronounced. The increase of α-Al makes it form a microgalvanic cell effect characterized by a “large cathode/small anode” configuration, accelerating the corrosion rate of η-Zn grains adjacent to α-Al. Wang et al. [28] reached the same conclusion in their research on the corrosion behavior of high-strength steel alloys. Therefore, corrosion prefers to extend along the extrusion direction with continuously distributed α-Al.

As shown in Figure 9e,f, the corrosion zone of filler metals can be divided into an intergranular corrosion area (ICA) and a uniform corrosion area (UCA). The width of the ICA in the Zn-2Al, Zn-2Al-0.3Ge, Zn-2Al-0.5, and Ge Zn-2Al-0.8Ge is 36 μm, 12 μm, 15 μm, and 32 μm. This shows that Ge can inhibit the generation of IGC along the extrusion direction. Ge facilitates the enrichment of precipitated Al in the filler metal, which increases the size of α-Al phases acting as cathodes. The increased size of the α-Al phase leads to a reduction in the Al content at the grain boundaries of the η-Zn phase. Liu et al. [14] also revealed that Ge decreases the densities of precipitates and improves the corrosion resistance of Al-Zn-Mg alloys. With increasing Al contents at the grain boundaries, the corrosion current decreases, and the anodic dissolution declines. However, it can be seen from Figure 9f–h that the width of the ICA rises from 12 μm to 32 μm as the Ge content increases from 0.3 wt.% to 0.8 wt.%, indicating the deterioration of corrosion resistance. In particular, the UCA appears for local IGC when the Ge content is 0.8 wt.%. The main reason for this is that excessive Ge will result in the precipitation of Ge phases at boundaries. The increase in Ge leads to the enrichment effect of Al at the grain boundary. The purification effect of Ge on grain boundaries is weakened. The Diamond-Ge and α-Al at boundaries form microcells with η-Zn, demonstrating the difficulties of corrosion generation along the η-Zn grain boundaries. At this time, the corrosion width of the filler metal decreases along the radial direction, and the corrosion resistance of the filler metal reduces.

**Figure 11:**
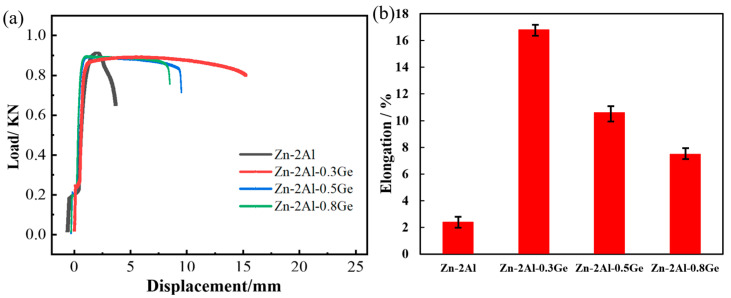
Mechanical properties of Zn-2Al-*x*Ge filler metals after 15 days of salt spray corrosion: (**a**) load–displacement curve and (**b**) elongation.

Figure 11 shows the load–displacement curve and elongation of Zn-2Al-*x*Ge filler metals after 15 d of salt spray corrosion. It can be seen that the displacement of four filler metals, from high to low, is as follows: Zn-2Al-0.3Ge > Zn-2Al-0.5Ge > Zn-2Al-0.8Ge > Zn-2Al. The Zn-2Al-0.3Ge filler metal has the greatest elongation (16%), which is greater than that of the Zn-2Al filler metal (2.4%) by 13.6%. This is mainly due to the regulatory effect of Ge on α-Al in the microstructure of filler metals. Ge promotes the enrichment of Al atoms in the filler metals, which decreases the Al content at the grain boundaries. The purity of grain boundaries is beneficial for suppressing intergranular corrosion. Therefore, Ge slows down the attenuation trend of the mechanical properties of filler metals that results from corrosion.

## 4. Conclusions

This study primarily investigates how minor Ge additions influence the corrosion behavior of Zn-2Al filler metals. The key conclusions are summarized as follows:

(1) The microstructure of Zn-Al-Ge filler metals mainly comprises three phases: η-Zn, α-Al, and Diamond-Ge. Ge reduces the free energy of the Zn-Al binary alloy system and promotes the enrichment of Al in the filler metal, which increases the size of α-Al phases.
(2) Under corrosive conditions, Zn-2Al filler metals undergo electrochemical corrosion, with α-Al and Diamond-Ge acting as cathodes and η-Zn as the anode. When the Ge content reaches 0.3 wt.%, the Al content at grain boundaries decreases, reducing the intergranular corrosion rate. Compared with the Zn-2Al filler metal, the elongation of the Zn-2Al-0.3Ge filler metal increases by 13.6% after 15 days of salt spray corrosion.
(3) When the Ge content in the filler metal rises to 0.8 wt.%, the purification effect of Ge on grain boundaries is weakened, and the corrosion resistance of Zn-2Al filler metals decreases.

## Figures and Tables

**Table 1:** Chemical composition of studied alloys (wt.%).

Filler Metal	Ge	Al	Mg	Cu	Mn	Zn
Zn-Al	0	1.95	0.20	0.16	0.20	Bal.
Zn-Al-0.3Ge	0.29	1.90	0.23	0.18	0.18	Bal.
Zn-Al-0.5Ge	0.48	1.97	0.19	0.16	0.20	Bal.
Zn-Al-0.8Ge	0.78	2.10	2.10	0.17	0.21	Bal.

**Table 2:** EDS results marked in Figure 1 (wt.%).

Point	Zn	Al	Ge
1	97.32	2.68	0
2	45.44	54.56	0
3	96.41	3.59	0
4	23.69	76.31	0
5	9.68	6.67	83.65

**Table 3:** Temperature parameters obtained from the DSC curve (°C).

Filler Metal	T_1_	T_eutectic1_	T_eutectic2_	T_proeutectic_	T_2_
Zn-Al	380.80	/	383.56	402.32	405.61
Zn-Al-0.3Ge	354.50	356.67	379.59	401.54	404.23
Zn-Al-0.5Ge	353.60	356.74	370.60	400.19	403.98
Zn-Al-0.8Ge	353.97	357.09	364.56	399.93	404.27

**Table 4:** EDS results marked in Figure 9 (wt.%).

Point	Zn	Al	Ge	O
6	50.32	12.01	0	37.67
7	5.77	81.20	0	13.03
8	31.43	53.47	0	15.00
9	0	1.05	94.93	4.02

## Data Availability

The original contributions presented in this study are included in the article. Further inquiries can be directed to the corresponding author.
